# Angiogenesis-Related Gene Expression Signatures Predicting Prognosis in Gastric Cancer Patients

**DOI:** 10.3390/cancers12123685

**Published:** 2020-12-08

**Authors:** Haoyu Ren, Jiang Zhu, Haochen Yu, Alexandr V. Bazhin, Christoph Benedikt Westphalen, Bernhard W. Renz, Sven N. Jacob, Christopher Lampert, Jens Werner, Martin K. Angele, Florian Bösch

**Affiliations:** 1 Department of General, Visceral, and Transplant Surgery, Ludwig-Maximilians-University Munich, D-81377 Munich, Germany; Haoyu.Ren@med.uni-muenchen.de (H.R.); Haochen.Yu@med.uni-muenchen.de (H.Y.); Alexandr.Bazhin@med.uni-muenchen.de (A.V.B.); Bernhard.Renz@med.uni-muenchen.de (B.W.R.); Sven.jacob@med.uni-muenchen.de (S.N.J.); Christopher.Lampert@med.uni-muenchen.de (C.L.); Jens.Werner@med.uni-muenchen.de (J.W.); martin.angele@med.uni-muenchen.de (M.K.A.); 2 Department of Endocrine and Breast Surgery, The First Affiliated Hospital of Chongqing Medical University, Chongqing 400016, China; 2013210399@stu.cqmu.edu.cn; 3 Department of Medicine 3 and Comprehensive Cancer Center, Ludwig-Maximilians-University Munich, D-81377 Munich, Germany; Christoph_Benedikt.Westphalen@med.uni-muenchen.de

**Keywords:** gene signature, angiogenesis, gastric cancer, nomogram, prognosis, biomarkers

## Abstract

**Simple Summary:**

To elucidate the role of angiogenesis as a prognostic signature in gastric cancer, we analyzed the expression level of 36 angiogenesis-related genes (ARGs) from Stomach Adenocarcinoma (STAD) from The Cancer Genome Atlas (TCGA). Consensus clustering analysis showed two major angiogenesis-related types: one related to more aggressive clinicopathological characteristics and worse survival, and the other related to lower tumor, lymph node, metastasis (TNM) stage and better outcomes. Our analysis of TCGA with a least absolute shrinkage and selection operator (LASSO) regression model identified 10 genes associated with overall survival in gastric cancer patients. With this gene signature, we computed angiogenesis-related gene signature risk scores for individual cancer patients that predicted overall and disease-free survival, which were further validated in the independent dataset Asian Cancer Research Group (ACRG). Moreover, an overall survival (OS)-related nomogram was established and had better performance in prognosis prediction than TNM stage. Our analysis provides a comprehensive map of ARGs that can be serve as useful biomarkers for gastric cancer.

**Abstract:**

Increasing evidence indicates that angiogenesis is crucial in the development and progression of gastric cancer (GC). This study aimed to develop a prognostic relevant angiogenesis-related gene (ARG) signature and a nomogram. The expression profile of the 36 ARGs and clinical information of 372 GC patients were extracted from The Cancer Genome Atlas (TCGA). Consensus clustering was applied to divide patients into clusters 1 and 2. Least absolute shrinkage and selection operator (LASSO) Cox regression analyses were used to identify the survival related ARGs and establish prognostic gene signatures, respectively. The Asian Cancer Research Group (ACRG) (*n* = 300) was used for external validation. Risk score of ARG signatures was calculated, and a prognostic nomogram was developed. Gene set enrichment analysis of the ARG model risk score was performed. Cluster 2 patients had more advanced clinical stage and shorter survival rates. ARG signatures carried prognostic relevance in both cohorts. Moreover, ARG-risk score was proved as an independent prognostic factor. The predictive value of the nomogram incorporating the risk score and clinicopathological features was superior to tumor, lymph node, metastasis (TNM) staging. The high-risk score group was associated with several cancer and metastasis-related pathways. The present study suggests that ARG-based nomogram could serve as effective prognostic biomarkers and allow a more precise risk stratification.

## 1. Introduction

Gastric cancer (GC) is a global health problem, with more than one million people newly diagnosed worldwide each year, almost two-thirds occurring in developing countries [1]. Most patients are diagnosed at advanced stage, even with distant metastasis. Although improvements in systemic therapy have been made, the mortality rate is still high, with five-year survival rates only around 30% worldwide [2]. In this respect, the initial response to anticancer treatment might diminish over time due to acquired resistance, representing a major multifactorial problem [3]. In essence, GC is a highly heterogeneous disease with different location types, histological types, molecular classifications, and biological behavior [4,5,6,7]. However, conventional risk assessment is mainly based on tumor, lymph node, metastasis (TNM) staging, which ignores the biological heterogeneity of the primary tumor. Therefore, it is critical to develop a multi-dimensional model to identify patients at high risk and aim to achieve personalized medicine in GC patients.

Genome analysis may offer new insights beyond TNM for characterizing tumor biology. In 2014, The Cancer Genome Atlas (TCGA) defined four distinct subtypes of stomach adenocarcinoma (Epstein–Barr virus (EBV) positive, microsatellite unstable (MSI), genomically stable (GS), chromosomal instability (CIN)) through comprehensive genomic profiling analysis [6]. This novel and innovative classification system described the genomic landscape of GC and provided a roadmap for patient stratification as well as a direction of targeted therapy. Nevertheless, TCGA typing based on European and American populations may not be applicable to Eastern populations. Therefore, selecting representative gene sets for tumor classification and developing predictive models can provide new ideas for more precise molecular subtypes and corresponding personalized therapy. Given the crucial role of angiogenesis in GC, it seems very promising to use angiogenesis related genes (ARGs) to provide effective risk stratification and identify potential targets for personalized therapeutic approaches. Angiogenesis is an essential process in tumorigenesis, because its induction is indispensable to deliver nutrients and evacuate metabolic waste [8]. During cancer development, several proangiogenic cytokines are released by tumor cells, such as vascular endothelial growth factor A (VEGFA), fibroblast growth factor (FGF) and hypoxia-inducible factor-1 (HIF-1), which contributes to the sprout and formation of neovasculature in the tumor microenvironment (TME) [9]. Thus, it had been demonstrated that anti-angiogenic therapies significantly improved prognosis in GC patients [10,11].

Based on comprehensive genome-wide gene expression profiles derived from TCGA, the present study aimed to develop prognostic relevant ARG expression signatures and a nomogram. These results were further validated in corresponding data from the Asian Cancer Research Group (ACRG).

## 2. Results

### 2.1. Cluster Analysis Based on ARG Expression Profiles

**Figure 1:**
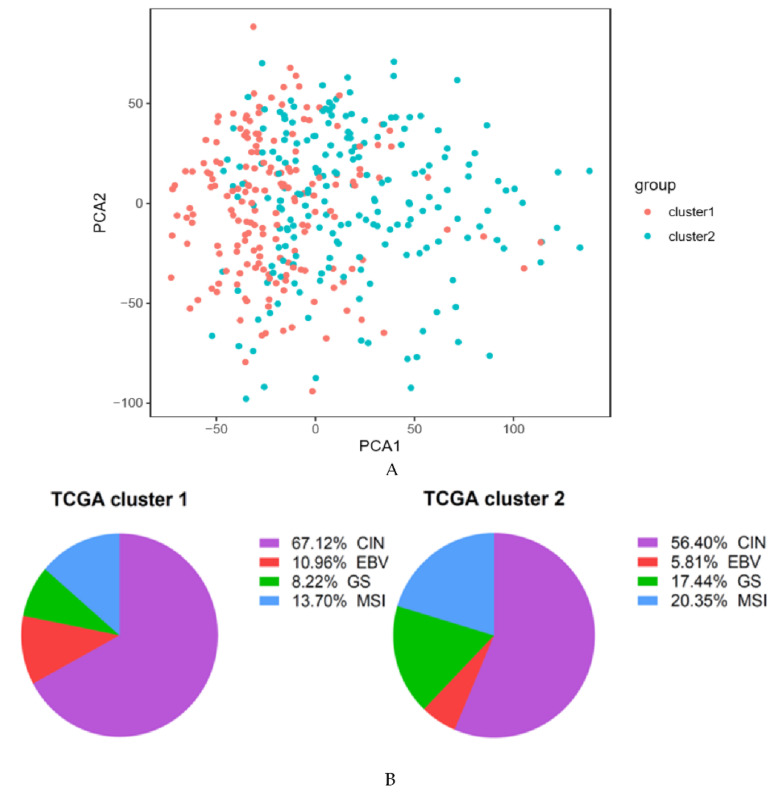
Two clusters based on the expression level of 36 angiogenesis-related genes (ARGs) in The Cancer Genome Atlas (TCGA) cohort. (**A**) Principal component analysis of the two clusters (cluster 1 and 2) in the TCGA cohort. (**B**) The proportion of different TCGA molecular subtypes in the two clusters. EBV: Epstein–Barr virus, MSI: microsatellite unstable, GS: genomically stable, CIN: chromosomal instability.

The ARG set was downloaded from “Gene Set Enrichment Analysis” (GSEA) (hallmark-angiogenesis [12], which includes 36 genes upregulated during the formation of tumorigenic blood vessels. To analyze the prognostic implication of ARGs in the training cohort (TCGA) of GC patients, a consensus clustering analysis was performed. As shown in Figure S1a–c, k = 2 was the optimal cluster number providing an excellent clustering stability in the training cohort. In Figure S1c, most of samples are concentrated on the far left, middle and far right. Density is too high to display every single sample. Therefore, the 372 GC patients from the training group were clustered into two subgroups (cluster 1 and 2). PCA was further applied to demonstrate the distinction of gene expression levels between the two subgroups, although the difference was not significant (Figure 1A). Meanwhile, the composition of the four different subtypes identified by TCGA in these two clusters was also analyzed (Figure 1B). Compared with cluster 2, the proportion of CIN and EBV-positive tumors in cluster 1 was higher, while GS and MSI subtypes were less.

**Figure 2:**
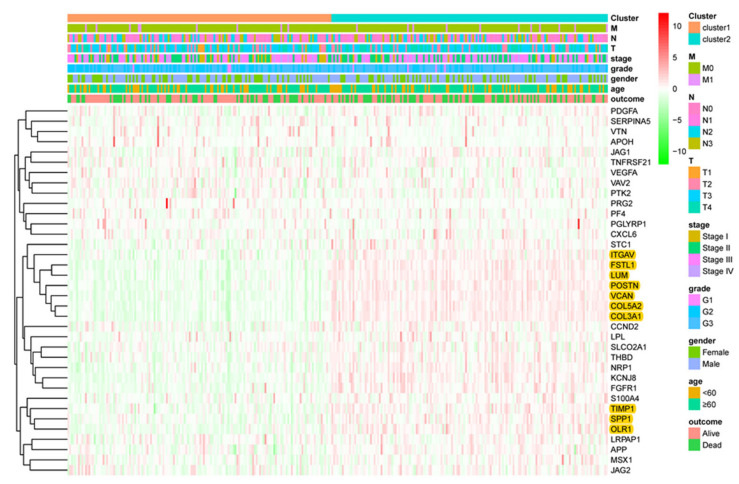
Heat map of clinicopathological features of the two subtypes (cluster 1 and 2). The overexpressed genes (ITGAV, FSTL1, LUM, POTN, VCAN, COL5A2, COL3A1, TIMP1, SPP1 and OLR1) in cluster 2 are highlighted. (T: primary tumor, T1: tumor invades the lamina propria, the muscularis mucosa, or the submucosa, T2: tumor invades muscularis propria layer, T3: tumor invades the subserosa layer without invasion of the serosa and adjacent structures, T4: tumor penetrates the serosa or adjacent structures, N: regional lymph node, N0: no regional lymph node metastases, N1: metastasis in 1-2 nodes, N2: metastasis in 3–6 nodes, N3: metastasis in more than 7 nodes, M: distant metastasis, M0: no distant metastasis, M1: distant metastasis).

Furthermore, the association between the clinicopathological characteristics and grouping was tested. Cluster 2 patients are more likely to have tumors with a higher grade (*p* = 0.004), a higher T stage (*p* = 0.002) and showed metastases significantly more often (*p* = 0.019). Gene expression analysis revealed that ITGAV, FSTL1, LUM, POTN, VCAN, COL5A2, COL3A1, TIMP1, SPP1 and OLR1 were overexpressed in cluster 2 patients compared to patients of cluster 1 (Figure 2). 

**Figure 3:**
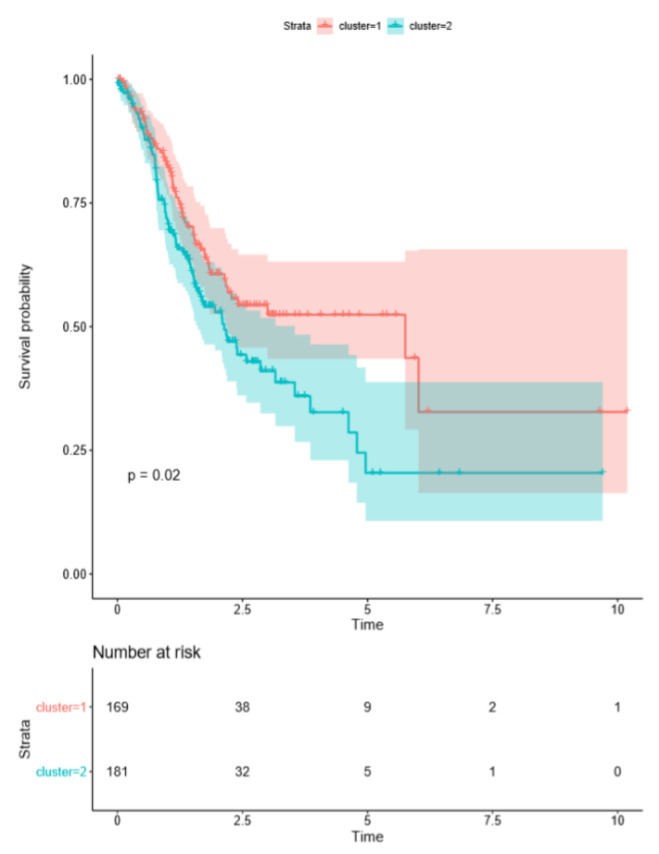
Kaplan–Meier survival curves for cluster 1 and 2 of the TCGA dataset. Overall survival of cluster 1 and cluster 2 (*p* = 0.02).

In this respect, the survival analysis showed that cluster 1 patients had significantly increased overall survival (OS) rates than patients of cluster 2 (*p* = 0.02; Figure 3). 

### 2.2. Identification of ARGs with Prognostic Value and Establishment of Prognostic Models

**Figure 4:**
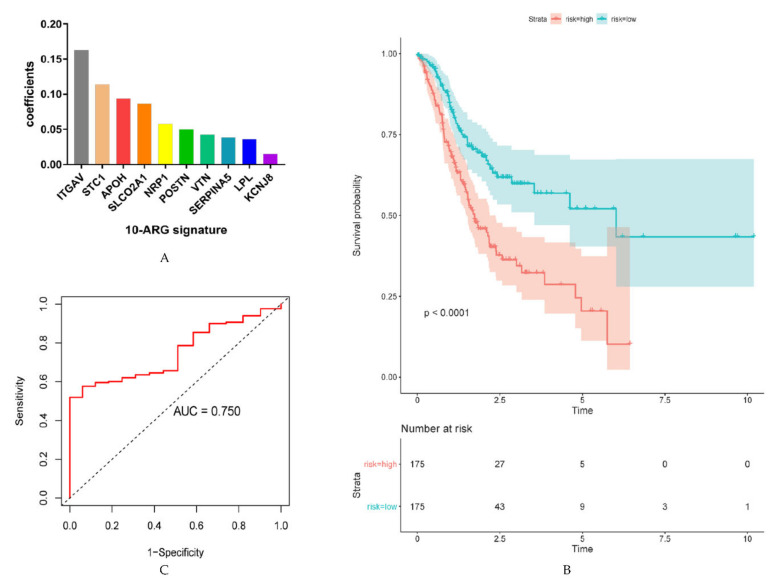
Development of overall survival (OS) prediction signature with angiogenesis related genes (ARGs). (**A**) The final 10 gene signatures with corresponding coefficients. (**B**) The survival analysis of the high- and low-risk score groups stratified based on the median of risk scores calculated by OS prediction risk score formula. (**C**) The receiver operating characteristic (ROC) curve for assessing the predictive ability of the 10-ARG signature.

In order to develop powerful predictive models based on ARGs, univariate Cox regression analysis was conducted. This analysis screened out 17 OS-related ARGs in the TCGA cohort (*p* < 0.05; Figure S2a). Subsequently, least absolute shrinkage and selection operator (LASSO) Cox regression analysis was performed to further analyze these 17 genes (Figure S2b,c). This analysis determined ten genes (ITGAV, STC1, APOH, SLCO2A1, NRP1, POSTN, VTN, SERPINA5, LPL, KCNJ8), which were used to build the prediction model (Figure 4A). Thus, the prediction risk score formula reads as follows: ITGAV×0.16310506+STC1×0.11382763+APOH×0.09369982+NRP1×0.05749847+POSTN×0.04985928+VTN×0.04265286+SERPINA5×0.03821238+LPL×0.03609897+KCNJ8×0.01472726. The prediction risk score of each TCGA patient was calculated, and patients in the training set were divided according to their risk score (median 7.239 in TCGA, median 1.483 in ACRG) into high- and low-risk groups. Kaplan–Meier survival analysis revealed that patients of the high-risk score group had highly significant shorter OS rates than those in the low-risk score group (*p* < 0.0001) (Figure 4B). Receiver operating characteristic (ROC) analysis of the predictive signature for 5-year OS showed an area under the curve (AUC) of 0.750 (Figure 4C). 

**Figure 5:**
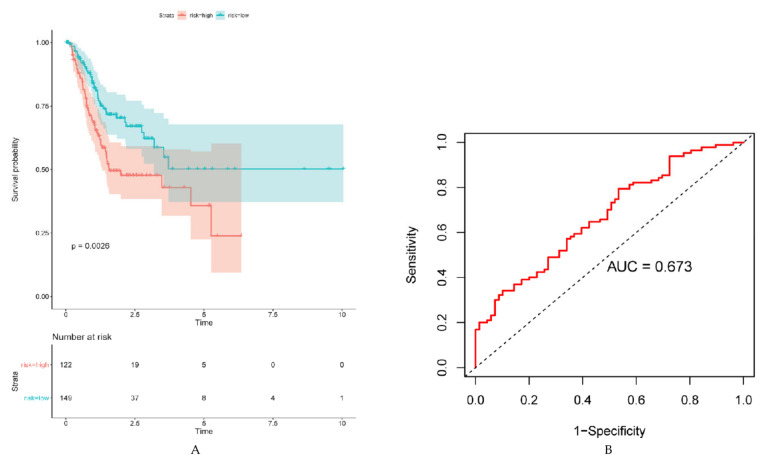
The performance of disease-free survival (DFS) in TCGA cohort based on the 10-ARG signature. (**A**) The DFS analysis of the high- and low-risk score groups stratified based on the median of risk scores calculated by 10-ARG signature prediction risk score formula. (**B**) The ROC curve for assessing the accuracy of DFS.

Furthermore, the above-mentioned prediction risk score was tested for its predictive value regarding disease-free survival (DFS) in the TCGA cohort. Figure 5A highlights DFS which proved to be highly significant different between high and low-risk score groups (*p* < 0.05). The ROC analysis of the ARG signature model for 2-year DFS revealed an AUC of 0.673 (Figure 5B).

### 2.3. Validation of Prognostic ARG Signatures with External Dataset

**Figure 6:**
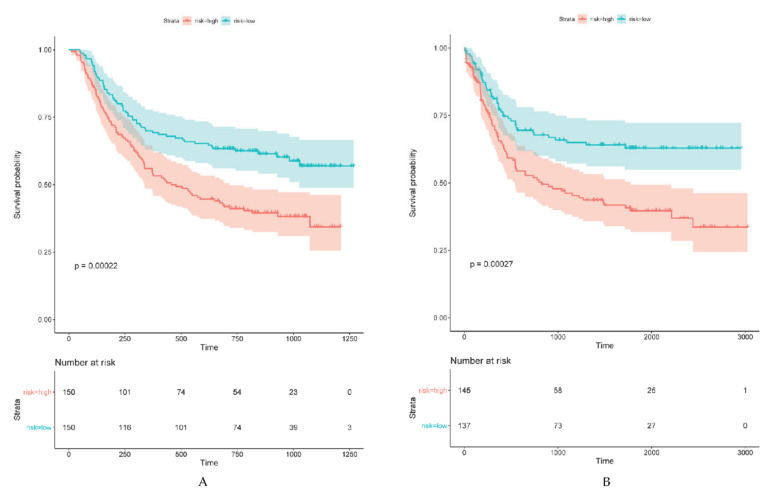
The prognostic performance of ARG signature risk score in the validation set. (**A**) Kaplan–Meier curve of overall survival of the ACRG cohort. (**B**) Kaplan–Meier curve of disease-free survival of the ACRG cohort.

To evaluate the prognostic power of the identified ARG signatures from the training data set, an independent dataset (ACRG cohort) was introduced as validation group. The established prediction risk score formula was used to calculate the risk score of each sample in the validation cohort. Similarly, the validation cohort was divided into high- and low-risk score groups using the corresponding median risk score as the cut-off value. As shown in Figure 6, the outcome of patients in the high-risk score group was significantly worse compared to patients in the low-risk score group. The risk score proved to be highly significant for OS (*p* < 0.001) (Figure 6A) and for DFS (*p* < 0.001) (Figure 6B).

### 2.4. ARG Signatures Independently Predict OS and DFS

To further confirm whether the newly generated risk score of the ARG signature was an independent risk factor in GC patients, various clinicopathological parameters were tested in both cohorts. In order to make the results easier to interpret and better present, patients were divided into two groups by cut-off points for age, stage and risk score based on previously published studies [13,14]. In the TCGA cohort, the univariate analysis revealed that age (*p* = 0.02), gender (*p* = 0.03), tumor stage (*p* = 0.004), and risk score (*p* < 0.001) were significantly associated with OS (Table 1a). Multivariate Cox regression analysis proved age, tumor stage, and risk score to be independent risk factors of OS (Table 1a). 

In addition, univariate and multivariate analyses indicated that a high-risk score was independently correlated with significantly poorer DFS (Table 1b). Consistent with the findings in the TCGA dataset, these were further validated by the ACRG cohort (Table 1c,d).

### 2.5. Construction and Validation of a Nomogram Based on ARG Signatures

**Figure 7:**
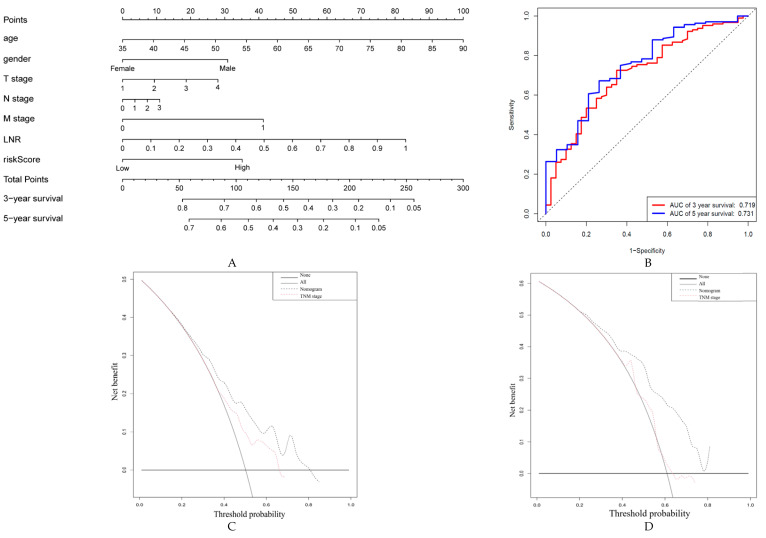
Establishment, assessment and validation of the nomogram to predict overall survival (OS) in gastric cancer patients. (**A**) The OS-related nomogram was developed in the TCGA cohort, with age, gender and T, N, M stage, lymph node ratio (LNR) and risk score incorporated. The different values for each parameter correspond to a point at the top of the axis. Points for all parameters are added and translated into the probability of 3- and 5-year survival. ROC curve of the OS-related nomogram at 3- and 5-year (**B**). Decision curve analysis of the OS-related nomogram at 3- (**C**) and 5-year (**D**). Net benefit of TNM staging alone and the combination of nomogram and TNM staging in making a more precise prediction of OS. “None” indicates that all samples were negative without intervention and the net benefit was 0. “All” indicates that all samples were positive with intervention.

To develop a clinically applicable tool easily assessing the prognosis of GC patients, a graphic nomogram was established. The nomogram was based on the training set predicting OS. The integrated clinicopathological features of the nomogram included age, gender, T stage, N stage, M stage, lymph node ratio (LNR) and the newly generated risk score (Figure 7A). ROC analysis and C-index were used to evaluate the prognostic value of the nomogram. The AUCs of the predictive value of the nomogram for the 3-, and 5-year OS in the TCGA dataset were 0.725 and 0.753, respectively (Figure 7B). The C-indexes of the nomogram in the training set and the validation set were 0.671 (95% CI; 0.62–0.73) and 0.704 (95% CI; 0.66–0.75), respectively. 

Additionally, the calibration plots showed a stable consistency between the nomogram-predicted probability and actual observation in terms of the 3- and 5-year OS in the TCGA cohort (Figure S3a,b). In addition, the nomogram was further tested in the validation group in terms of the calibration plots. This analysis demonstrated a significant correlation with the training results (Figure S3c,d). 

Decision curve analysis (DCA) of the nomogram was performed in the TCGA cohort and demonstrated that the nomogram model had an excellent net benefit for 3- and 5-year OS (Figure 7C,D). Compared to the conventional TNM staging system, the nomogram built with the ARG signature risk score had a better performance in predicting OS. Moreover, the comparison between the nomogram with the ARG risk score and a nomogram that only contained the clinicopathologic features was also conducted. Compared with the nomogram with only clinicopathological factors, the ARG risk score-based nomogram has better discrimination and calibration (Figure S4a–c). Moreover, the nomogram based on the risk score slightly added more net benefit than the one without the risk score or the model based on clinicopathological factors, and the threshold probability ranged from 0.6 to 0.75 (Figure S3e,f). Therefore, these results showed that the nomogram based on ARGs risk score can be used as an effective method to predict prognosis of patients in clinical practice.

### 2.6. Functional Analysis of the ARG Signatures

**Figure 8:**
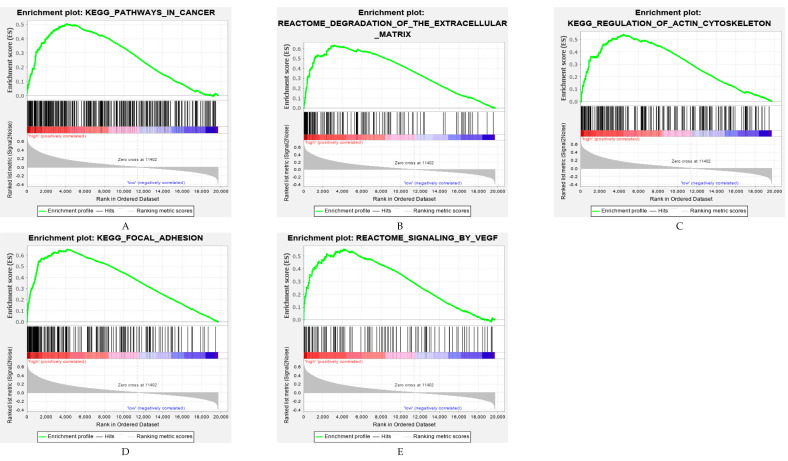
Gene set enrichment analysis identified biological pathways and processes associated with a high-risk score within the TCGA cohort. (**A**–**E**) Selected enrichment plots of canonical pathway gene sets between overall survival-related high- and low-risk score groups based on the 10-ARG signature prediction model.

To elucidate the potential influence of the ARG-related classifier on the expression profiles of GC, GSEA was applied to compare the high- and low-risk groups. Based on the OS-related ARG signature risk score, the gene sets of the high-risk group were mainly enriched in cancer- and metastasis-related pathways, including KEGG (pathways in cancer, regulation of actin cytoskeleton and focal adhesion) and REACTOME (pathways of degradation of the extracellular matrix and signaling by VEGF) (Figure 8A–E).

## 3. Discussion

Substantial evidence suggests that angiogenesis is involved in processes of carcinogenesis, progression, and metastasis of GC. Moreover, results from translational research on angiogenesis in GC indicate that several angiogenesis-related factors might be prognostically relevant [9,15,16,17]. Although analyzing the expression levels of a single angiogenetic gene is convenient with immunohistochemistry and ELISA [17,18], multiple gene signature analysis reflects the complex interaction of various parameters affecting angiogenesis in tumor pathology. Therefore, this multigene approach might allow the characterizing of tumor biology, thereby supporting clinical decision-making in times of cancer precision medicine.

In this study, consensus clustering according to the expression levels of 36 ARGs identified two innovative subtypes, clusters 1 and 2, significantly associated with clinicopathological features. However, the PCA showed that there is not a clear difference between the two clusters. The main reason is that this algorithm may miss some information as compared to the original features. Moreover, the principal component is obtained by dimension reduction, which leads to a negligible loss of information during the analysis process. The TCGA molecular subtypes were analyzed according to the clustering. This analysis revealed that cluster 2 patients more often had GS and MSI tumors, but fewer EBV associated carcinomas. GS and MSI tumors are characterized by several important molecular alterations, such as RHOA (Ras Homolog Family Member A) mutation, CLDN18-ARHGAP26 fusion, PIK3CA and EGFR (Epidermal Growth Factor Receptor) mutations [19]. In cluster 2, GC expressed higher levels of ARGs, therefore it can be inferred that anti-angiogenesis therapy might have a better response to GS and MSI subtypes, which needs to be confirmed in future studies. Most importantly, in both cohorts (TCGA, ACRG) a significant difference in overall survival was evident between the two novel subtypes. Moreover, gene sets closely related to the high-risk group were further explored, which might help to understand the poor prognosis of patients in the high-risk group. Nonetheless, because this study focused on ARGs, this pre-selection introduced a bias to GSEA. The present study revealed that the expression profiles of the high-risk score group significantly correlated with an increased expression of metastasis-related processes, such as degradation of the extracellular matrix (ECM), focal adhesion and VEGF signaling [20,21]. In this respect, a significant relationship between the immune microenvironment and pathological angiogenesis in GC has previously been reported [22]. The ECM is a key component of the tumor and cancer progression, due to it acting on endothelial cells [23]. Besides, integrin-mediated adhesion plays an important role during angiogenesis and protects the integrity of endothelial cells [24]. Thus, the identified ARG pathways add information on tumor biology further characterizing gastric cancer. 

In addition to the above-mentioned gene clusters, an ARG-based risk score has been established. Five (ITGAV, POSTN, VTN, STC1, NRP1) of the identified genes were previously investigated in GC. The present findings suggest that ITGAV was the main contributing gene because of the highest coefficient. Wang et al. showed that ITGAV was highly expressed in GC, which was associated with advanced tumors and deteriorated survival rates [25]. Moreover, high ITGAV expression rates correlated with deteriorated survival rates in breast cancer [26], liver cancer [27] and osteosarcoma [28]. Meanwhile, an ITGAV antagonist (cilengitide) was proven to inhibit angiogenesis and metastasis in breast cancer [26]. 

As mentioned above, the ARG signature correlated with pathways associated to the degradation of ECM. The ARG signature included two ECM related genes (VTN, POSTN) and high expression rates of these genes are associated with worse outcome in multiple malignancies [29,30]. VTN, as a downstream target of VEGFR2, has been reported to be related to promoting the metastasis and proliferation of GC [31]. POSTN binds to integrins and by promoting adhesion and migration of epithelial cells it is involved in metastasis formation and further supports invasion of GC cells [18]. 

Furthermore, two genes (STC1, NRP1) encoding glycoproteins are also included in the ARG signature. STC1, involved in various cancer-related signaling pathways [32], induces VEGF expression in GC cells [17] and NRP1 induces proliferation, migration, and metastasis of GC cells [33]. Additionally, in patients with GC, the overexpression of STC1 was significantly associated with higher proliferation rates, chemoresistance, metastasis formation, and deteriorated survival rates [34]. More importantly, STC1 and NRP1 are both connected with the VEGF/VEGFR2 pathway [35,36]. In this regard, the present results suggest that the identified ARG signature was closely related to a higher malignancy of GC. Therefore, the ARG signature might be an easily applicable tool supporting clinical decision-making. 

In the present study, the newly established risk score was an independent prognostic factor in multivariate analysis. The AUCs of the ARG signature was 0.750. Previously published gene expression-based scores for the prognosis of GC only reached an AUC of 0.671 [37]. Notably, to establish prediction signatures and models, which provided more insightful prognosis analyses than other studies, two databases with complete clinical follow-ups were used in the present analysis. Additionally, the evaluated model proved to be significant in both the training and the validation cohort. Thus, the present study provides an additional tool in achieving a more precise diagnosis and could provide support in treatment decision making.

In an attempt to evaluate the ARG-based risk score in a clinical setting, a nomogram was established. Nomograms combine multiple prognostic significant factors, and thus nomograms have become a powerful and easy-to-use tool to assess the survival probability of cancer patients [38,39]. Building a nomogram can transform statistical predictive models into a single numerical estimate of a patient’s outcome, which is tailored to the background of each patient. The nomogram of the present study, combining age, gender, TNM stage, LNR and ARG risk score, yielded a favorable predictive performance. Out of the included parameters, age, was the most significant prognostic marker. A Surveillance, Epidemiology, and End Results (SEER) data-based study showed that younger GC patients had improved survival rates after surgery than elderly patients [40]. This might be in part due to the fact that younger patients had a better tolerance to surgery, chemotherapy, and recovered faster [41]. LNR was also introduced into the two nomograms, which had been proposed as a sophisticated prognostic marker reflecting the quantity of metastatic lymph nodes as well as the quality of lymph node dissection [42]. It has been proven that LNR can accurately identify patients at high-risk of recurrence [43]. The TNM staging system represents the standardized benchmark to categorize patients with GC, evaluate prognosis, and recommend the optimal treatment [44]. The impact of tumor heterogeneity on individual prognoses is still difficult to assess. Thus, the addition of the ARG risk score made the nomogram more reliable because it was associated with outcome in both training set and validation set. Although the increase in net benefit was not clearly obvious compared to the nomogram without the risk score, the ARG risk score-based nomogram had a better performance in discrimination and calibration. Thus, the present findings suggest that the established nomogram has a better predictive value than the current TNM staging system and the nomogram that excludes the ARG risk score. The threshold probability ranged from 0.6–0.75 and the ARG-based nomogram was superior to a model based on clinicopathological features alone. In this respect, the three-year and five-year survival rates in the TCGA-STAD cohort were 60.8% and 58.8%, respectively. Therefore, the present findings suggest that the predictive value of the ARG risk score model might be beneficial regarding the three-year survival rate of GC patients compared to the model excluding the risk score. However, this difference is not consistent regarding the five-year survival rate. 

To the best of our knowledge, this is the first and most comprehensive study identifying prognosis related ARGs and developing prognostic relevant nomograms in patients with GC. However, there are limitations as well. Firstly, this was a retrospective study for the establishment of gene signatures based on public databases [6,7]. Moreover, TCGA is the world’s largest and richest collection of genomic data. The clinical data and genomic information are comprehensive and reliable. It also contains the gastric cancer database with the largest sample size. Nonetheless, these databases are well characterized, and the signatures proved a significant benefit in both cohorts. Secondly, this analysis has been conducted in silico, introducing a bias. Correction for multiple testing has not been conducted, therefore the results of the present study have to be interpreted with caution. This theoretical approach which seems to provide an additional benefit should be further tested in a well characterized prospective collective. Furthermore, subsequent studies should focus on information about systemic treatment, response rates, and acquired resistance to therapy. In particular, resistance represents a major multifactorial problem leading to deteriorated survival rates [3].

## 4. Materials and Methods 

### 4.1. Gene Expression and Clinical Data Acquisition

The level III gene expression profiles and corresponding clinical information, such as TNM classification, age, gender, overall survival, of patients with stomach adenocarcinoma were downloaded from the TCGA data portal as a training set, which contained 372 stomach adenocarcinoma samples and 35 adjacent normal tissues after excluding incomplete cases. The disease-free survival (DFS) rates of the TCGA STAD (stomach adenocarcinoma) cohort were obtained from the cBio Cancer Genomics Portal [45]. 

As a validation set, data of 300 GC patients from ACRG were downloaded, which included gene expression data and follow-up information (overall survival (OS) and DFS). All gene expression data were log2-transformed.

### 4.2. Consensus Clustering Analysis

In order to investigate the function of ARGs in GC, we divided tumor samples into different clusters with “ConsensusClusterPlus” (50 iterations, resample rate of 80%). Thereafter, PCA (principal component analysis) was used to validate the reliability of clustering with the R package “ggplot2”. PCA (principal component analysis) was used to validate the reliability of clustering with the R package “ggplot2”. Heatmaps were generated using the package “pheatmap” in the statistical programming language R (developed by RC Team, Vienna, Austria) [46].

### 4.3. Development and Validation of Prognostic Signatures Based on ARGs

The OS-related ARGs found to be statistically significant in univariate Cox regression analysis were then used in least absolute shrinkage and selection operator (LASSO) regression analysis with the R package “glmnet”. In order to prevent overfitting effects of the model, the penalty regularization parameter λ was determined via the ten-fold cross validation. Ten ARGs were selected to build the risk signature based on the optimal lambda value and the corresponding coefficients. The risk score of ARG signature for each patient was calculated as follows:

(1) $$Risk\;score=\overset{n}{\underset{i=1}{\sum }}(Exp_{i}*\beta_{i})$$

where n is the number of selected ARGs, $Exp_{i}$ is the expression value of gene i, and $\beta_{i}$ is the coefficient of gene i generated from LASSO regression analysis. All patients were divided into high- and low-risk groups by the median risk score.

Finally, Kaplan–Meier analysis was used to evaluate the differences of OS and DFS between high- and low-risk groups in the two cohorts. This analysis was performed with R software based on the R package “survival” and “survminer”.

### 4.4. Construction and Evaluation of the Nomogram

A nomogram and calibration plots were established by utilizing the “rms” package in R software. The time-dependent receiver operating characteristic (ROC) curves were used to determine the prognostic performance of the gene signature and nomogram model with R package “pROC”. The calculation of Concordance index (C-index) is to estimate the probability that the predicted result is consistent with the actual outcome. Calibration curves were plotted to assess the discrimination of the nomogram and the 45° dotted line indicates the optimal prediction. In addition, decision curve analysis (DCA) was performed to evaluate the clinical usefulness and to compare the established nomogram with the conventional TNM staging system and the nomogram without the ARG risk score.

### 4.5. Gene Set Enrichment Analysis (GSEA)

GSEA was used to identify the expression of differentially expressed gene sets between high- and low-risk score groups of the two ARG signatures through MSigDB C2 CP: Canonical pathways gene set collection (1485 gene sets available). GSEA was conducted by the JAVA program [12]. Gene set permutations were conducted 1000 times for each evaluation. 

### 4.6. Statistical Analysis

Statistical analyses were performed using R software v4.0.0 (R Foundation for Statistical Computing, Vienna, Austria). All tests were two-tailed and *p*-values < 0.05 were considered statistically significant and *p*-values < 0.001 were considered highly significant. FDR (false discovery rate) *q* < 0.05 was considered statistically significant.

## 5. Conclusions

In conclusion, the present study demonstrated that the identified ARG signature was a reliable prognostic and predictive marker for OS and DFS in patients with GC. Furthermore, the ARG-based risk score and the nomogram were independent prognostic factors. These additional and easily usable tests might facilitate personalized treatment and guide clinical decisions. In addition, an ARG-based stratification of patients with gastric cancer might improve the value of clinical trials. 

## Figures and Tables

**Table 1:** Independent prognostic factors for overall and disease-free survival in gastric cancer patients. Univariate and multivariate Cox regression analysis of the relationship between clinicopathological features (including the risk score) and overall survival and disease-free survival of patients in the TCGA (a and b) and ACRG (c and d) datasets.

Variable	Overall Survival in TCGA			
	Univariate Analysis		Multivariate Analysis	
	Hazard Ratio (HR) (95%CI)	*p*-Value	HR (95%CI)	*p*-Value
Age (≥60 vs. <60 years)	1.63 (1.10−2.42)	0.02	1.62 (1.09−2.41)	0.02
Gender (male vs. female)	1.51 (1.03−2.21)	0.03	1.43 (0.95−2.04)	0.09
Stage (III+IV vs. I+II)	1.70 (1.18−2.43)	0.004	1.64 (1.14−2.35)	0.008
Risk score (high vs. low)	1.99 (1.39−2.83)	<0.001	1.84 (1.29−2.63)	<0.001
(a)				
**Variable**	**Disease-Free Survival in TCGA**			
	**Univariate Analysis**		**Multivariate Analysis**	
	**HR (95%CI)**	***p*-Value**	**HR (95%CI)**	***p*-Value**
Age (≥60 vs. <60 years)	0.97 (0.64−1.47)	0.89	1.10 (0.72−1.67)	0.67
Gender (male vs. female)	1.82 (1.14−2.91)	0.01	1.76 (1.10−2.81)	0.02
Stage (III+IV vs. I+II)	1.44 (0.96−2.17)	0.08	1.37 (0.91−2.07)	0.13
Risk score (high vs. low)	1.98 (1.32−2.98)	<0.001	1.81 (1.21−2.72)	0.004
(b)				
**Variable**	**Overall Survival in ACRG**			
	**Univariate Analysis**		**Multivariate Analysis**	
	**HR (95%CI)**	***p*-Value**	**HR (95%CI)**	***p*-Value**
Age (≥60 vs. <60 years)	1.26 (0.89−1.77)	0.19	1.58 (1.11−2.24)	0.01
Gender (male vs. female)	0.92 (0.66−1.28)	0.61	0.88 (0.63−1.23)	0.45
Stage (III+IV vs. I+II)	3.41 (2.34−4.97)	<0.001	3.23 (2.21−4.72)	<0.001
Risk score (high vs. low)	1.83 (1.32−2.54)	<0.001	1.72 (1.23−2.41)	0.002
(c)				
**Variable**	**Disease-Free Survival in ACRG**			
	**Univariate Analysis**		**Multivariate Analysis**	
	**HR (95%CI)**	***p*-Value**	**HR (95%CI)**	***p*-Value**
Age (≥60 vs. <60 years)	1.09 (0.76−1.55)	0.64	1.33 (0.84−2.12)	0.23
Gender (male vs. female)	0.98 (0.68−1.42)	0.93	0.87 (0.54−1.40)	0.56
Stage (III+IV vs. I+II)	4.07 (2.62−6.33)	<0.001	3.76 (2.41−5.85)	<0.001
Risk score (high vs. low)	1.95 (1.35−2.80)	<0.001	1.68 (1.15−2.44)	0.007
(d)				

## Supplementary Materials

The following are available online at https://www.mdpi.com/2072-6694/12/12/3685/s1, Figure S1: Consensus clustering analysis of the angiogenesis related genes in the TCGA cohort. Figure S2: Univariate Cox and LASSO Cox regression analysis for OS-related ARGs. Figure S3: The calibration plots and decision curve analysis of the OS-related nomogram in two datasets. The calibration plots for predicting OS at 3. Figure S4: The discrimination and calibration of the nomogram without ARG risk score. ROC curve of the nomogram only contains clinicopathological features at 3- and 5-year.
