# Supplementary material for: Angiogenesis-Related Gene Expression Signatures Predicting Prognosis in Gastric Cancer Patients
Source: Cancers (Basel). 2020 Dec 8;12(12):3685. doi: 10.3390/cancers12123685 (PMC7763234; doi:10.3390/cancers12123685)
Supplement: Supplementary file 1 [file cancers-12-03685-s001.pdf]

cancers

Supplementary materials

Angiogenesis-Related Gene Expression Signatures Predicting Prognosis in Gastric Cancer Patients

Haoyu Ren [1], Jiang Zhu [2], Haochen Yu [1], Alexandr V. Bazhin [1], C. Benedikt Westphalen [3], Bernhard W. Renz [1], Sven N. Jacob [1], Christopher Lampert [1], Jens Werner [1], Martin K. Angele [1] and Florian Bösch [1,*]

1 Department of General, Visceral, and Transplant Surgery, Ludwig-Maximilians-University Munich, D-81377 Munich, Germany; Haoyu.Ren@med.uni-muenchen.de (H.R.); Haochen.Yu@med.uni-muenchen.de (H.Y.); Alexandr.Bazhin@med.uni-muenchen.de (A.V.B.); Bernhard.Renz@med.uni-muenchen.de(B.W.R.); Sven.jacob@med.uni-muenchen.de (S.N.J.); Christopher.Lampert@med.uni-muenchen.de (C.L.); Jens.Werner@med.uni-muenchen.de (J.W.); martin.angele@med.uni-muenchen.de (M.K.A.);
2 Department of Endocrine and Breast Surgery, The First Affiliated Hospital of Chongqing Medical University, 400016 Chongqing, China; 2013210399@stu.cqmu.edu.cn
3 Department of Medicine 3 and Comprehensive Cancer Center, Ludwig-Maximilians-University Munich, D-81377 Munich, Germany; Christoph_Benedikt.Westphalen@med.uni-muenchen.de
* Correspondence: Florian.Boesch@med.uni-muenchen.de; Tel.:+49-89-4400-72781; Fax: +49-89-4400-75474

Received: 30 September 2020; Accepted: 4 December 2020; Published: 8 December 2020

Supplementary

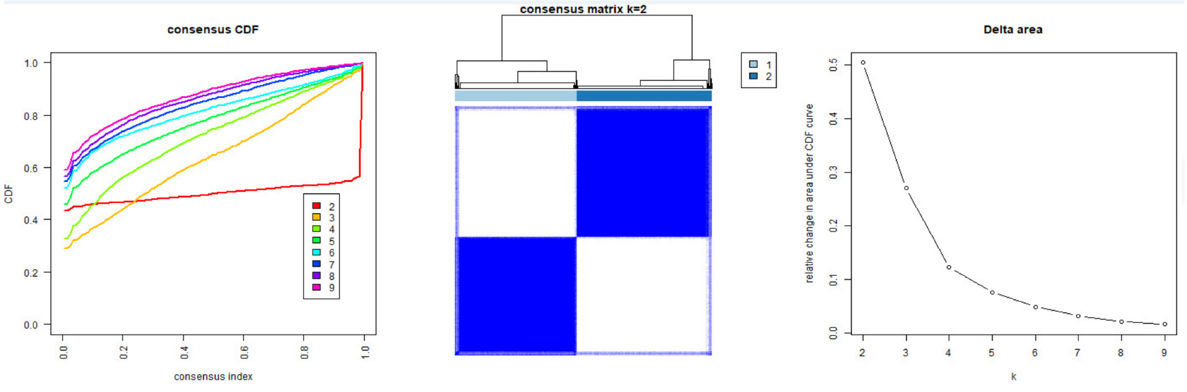

A B C

Figure S1. Consensus clustering analysis of the angiogenesis related genes in the TCGA cohort. (a) Consensus clustering cumulative distribution function (CDF) with k valued 2 to 9. (b) Relative change in area under the CDF curve with k valued 2 to 9. (c) Consensus clustering matrix for k = 2.

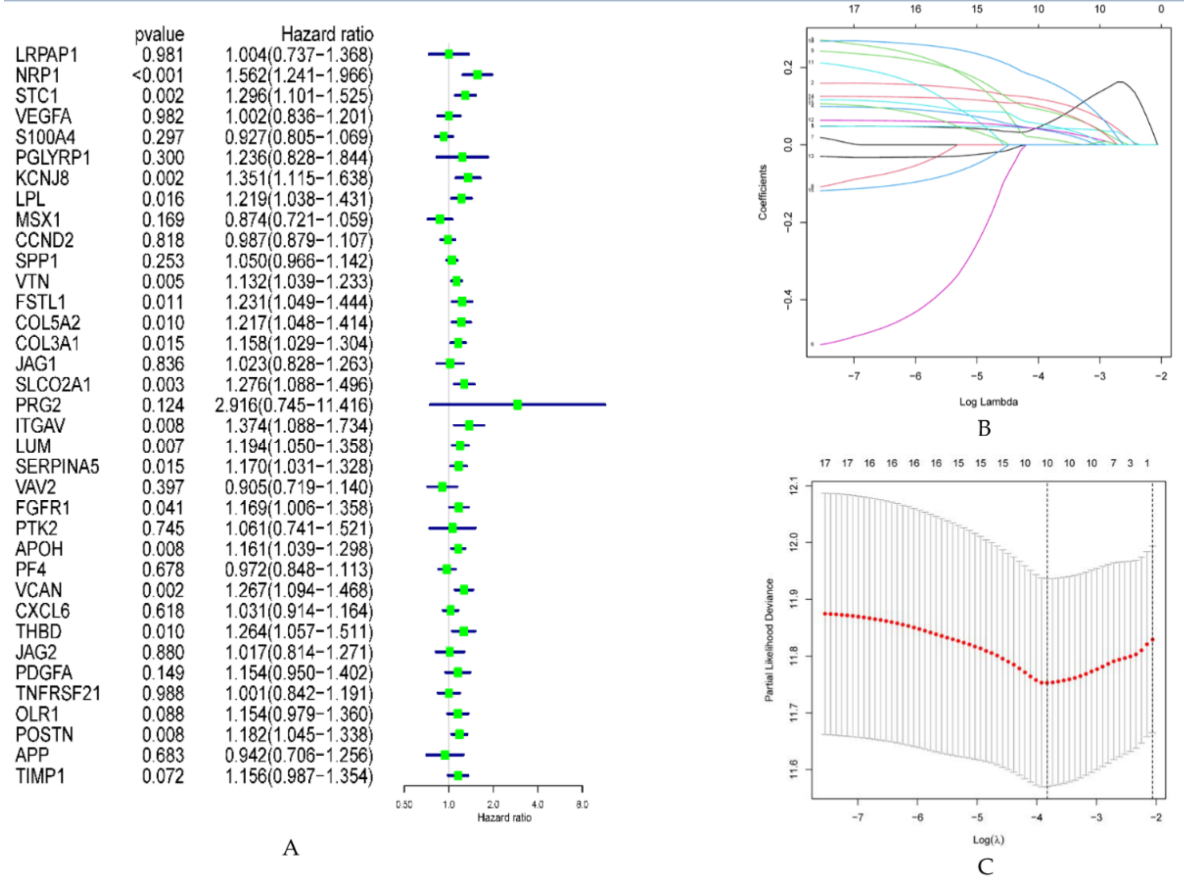

Figure S2. Univariate Cox and LASSO Cox regression analysis for OS-related ARGs. (a) Univariate Cox analysis of the 36 ARGs derived from the TCGA dataset in terms of OS. (b, c) LASSO Cox regression analysis of the selected 17 ARGs.

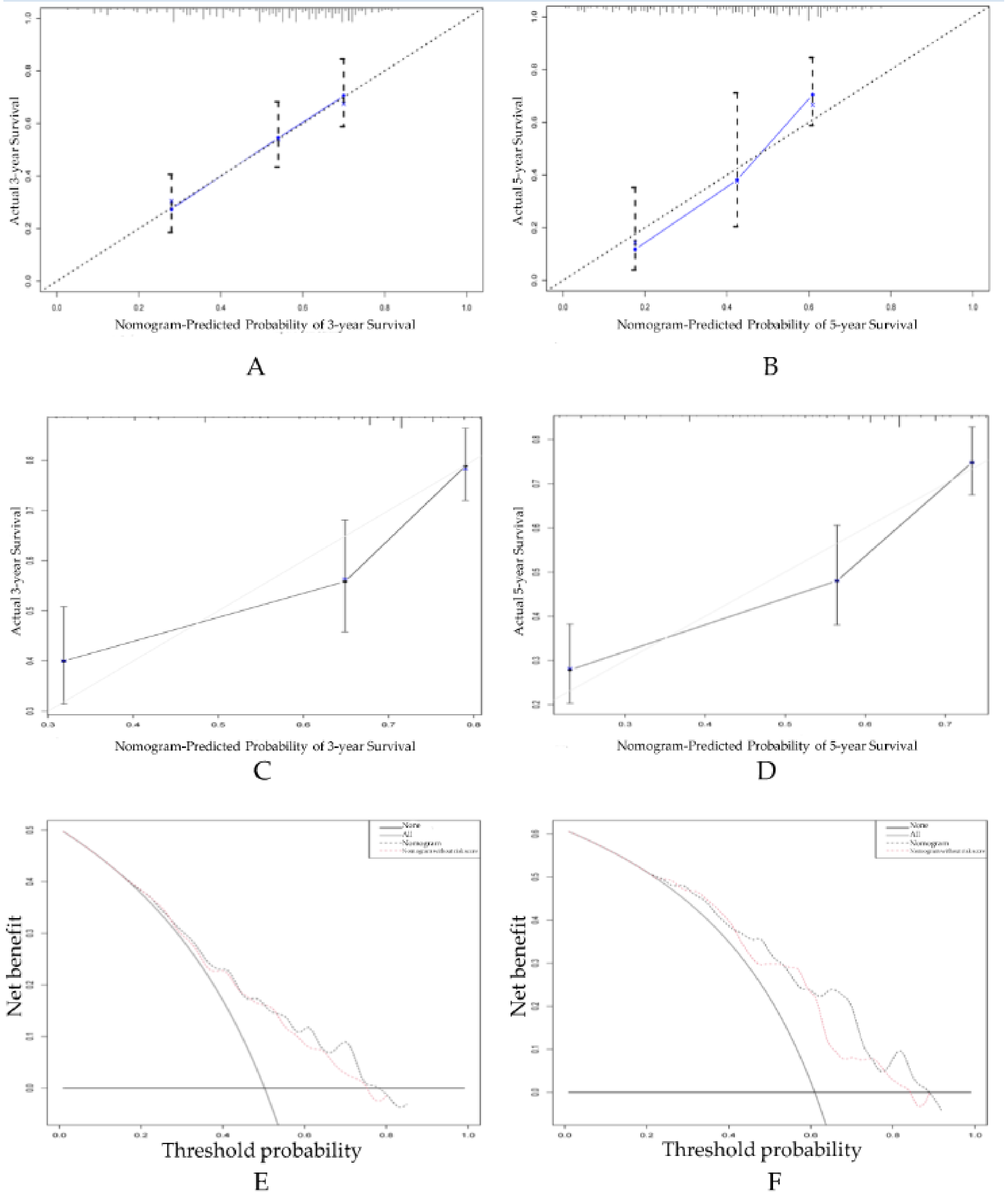

Figure S3. The calibration plots and decision curve analysis of the OS-related nomogram in two datasets. The calibration plots for predicting OS at 3- (a) and 5-year (b) in the training set and the calibration plots for predicting OS at 3- (c) and 5-year (d) in the validation set. Nomogram-predicted survival is plotted on the x-axis, actual survival is plotted on the y-axis. The line at 45° represents ideal prediction. Vertical lines represent 95% confidence intervals (CI). Decision curve analysis of the OS-related nomogram at 3- (e) and 5-year (f) in the TCGA cohort. Net benefit of the nomogram that only contains the clinicopathologic features and the combination of nomogram and ARG risk score in making a more precise prediction of OS. “None” indicates that all samples were negative without intervention and the net benefit was 0. “All” indicates that all samples were positive with intervention.

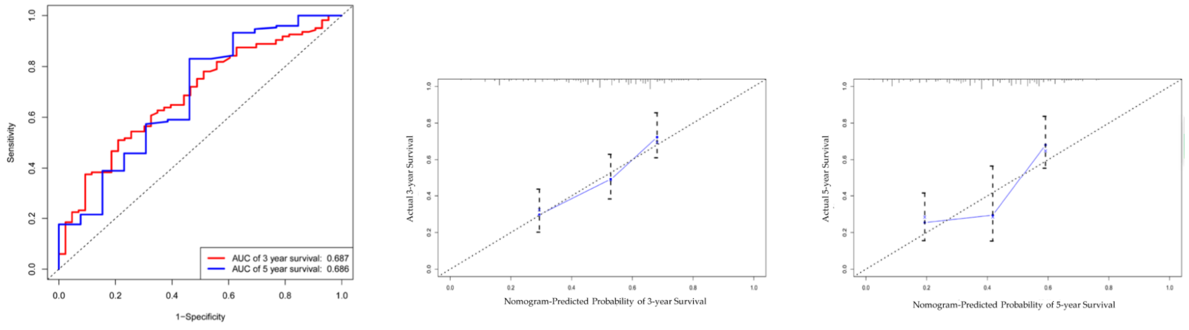

A B C

Figure S4. The discrimination and calibration of the nomogram without ARG risk score. ROC curve of the nomogram only contains clinicopathological features at 3- and 5-year (a). The calibration plots for predicting OS at 3- (b) and 5-year (c) in the TCGA cohort. Nomogram-predicted survival is plotted on the x-axis, actual survival is plotted on the y-axis. The line at 45° represents ideal prediction. Vertical lines represent 95% CI.
